# Drug-Induced Liver Injury Secondary to Terbinafine Use

**DOI:** 10.7759/cureus.54453

**Published:** 2024-02-19

**Authors:** Aaron Brown, Mark Potesta, Ajmal Hameed

**Affiliations:** 1 Medicine, Lake Erie College of Osteopathic Medicine, Bradenton, USA; 2 Gastroenterology, HCA Florida Memorial Hospital, Jacksonville, USA

**Keywords:** acute liver failure (alf), liver toxicity, drug-induced liver injury (dili), liver hepatitis, terbinafine

## Abstract

Drug-induced liver injury (DILI) has a symptomatic profile that mimics many forms of hepatic injury. In patients presenting with symptoms suspicious of acute liver injury, it is important that clinicians effectively rule out more common causes while simultaneously maintaining a broad differential diagnosis that includes DILI. In this report, we present the case of a 41-year-old African American male who was admitted to the hospital for two weeks’ duration of worsening jaundice, right upper quadrant pain, pruritus, and acholic stools after terbinafine use for an acute episode of onychomycosis. Physical examination showed evidence of jaundice, scleral icterus, and a soft non-distended abdomen. Initial laboratory results at admission showed significant elevation of total bilirubin, alkaline phosphatase, aspartate aminotransferase, and alanine aminotransferase. Careful review of the patient’s medications, a clinical workup to rule out primary causes of hepatobiliary pathology, and confirmatory liver biopsy showing benign hepatic parenchyma with marked cholestasis including bile plugs and bile granulomas provided sufficient evidence supporting terbinafine use as the inciting factor. The emphasis of this case is to highlight the symptoms, diagnostic measures, and suspected pathophysiology of terbinafine-induced hepatotoxicity.

## Introduction

Terbinafine is an orally and topically active allylamine antifungal agent that is used to treat superficial infections of the skin and nails [1]. Terbinafine is a fungicidal agent that, when given orally, is the first-line treatment against mild-to-moderate dermatophyte onychomycosis [2]. Terbinafine works through selective inhibition of fungal squalene epoxidase, raising levels of squalene to toxic levels and thus eliminating the infection [1]. In the oral form, when treating dermatophyte fingernail infection, it is typically given as a 250-mg six-week course. When treating toenail infection, the same dose is used; however, the treatment course typically spans 12 weeks [1]. Although terbinafine has shown excellent efficacy in treating onychomycosis, the drug maintains a wide-ranging side effect profile. Frequently, patients may experience a combination of typical, self-remitting side effects such as gastrointestinal disturbances, headaches, changes in taste, and/or rash [1]. Rarely, terbinafine may cause a potentially fatal drug-induced liver injury (DILI) [1]. DILI is an adverse effect of medication use that can result in an acute episode of hepatitis or cholestasis. Common symptoms of DILI can include jaundice, scleral icterus, influenza-like symptoms, dark urine, or pruritus. Most cases of DILI are self-limited; however, some require discontinuation of medication for resolution of symptoms [1,2]. Oral therapy of terbinafine is associated with mildly elevated serum aminotransferase levels in less than 1% of patients. In these patients, the findings are typically subclinical, not necessitating a halt in treatment. The estimated probability of developing elevated serum aminotransferase level requiring a stop in treatment is 0.31% for two to six weeks of treatment and 0.44% for treatment longer than eight weeks [3]. Most commonly, liver injury arises within the first six weeks of therapy. Furthermore, estimated hepatic injury may be described as one in 50,000 to 120,000 prescriptions [1]. The pattern of injury typically varies initially, either cholestatic (with elevation in bilirubin and alkaline phosphatase [ALP]) or hepatocellular (with elevations in aspartate aminotransferase [AST] and alanine aminotransferase [ALT]). Most frequently, the pattern of terbinafine-induced liver injury evolves into a cholestatic pattern [4].

## Case presentation

A 41-year-old African American male with a history significant for type 2 diabetes mellitus and current incarceration was admitted to the hospital for two weeks’ duration of worsening jaundice, right upper quadrant pain, pruritus, and acholic stools. The patient denied any history of liver disease. He admitted to a history of alcohol use and to being a previous 10-pack-year cigarette smoker before stopping three years prior to presentation. He denied the use of any recreational drugs or any recent tylenol use. The patient revealed a recent history of terbinafine use for an acute episode of onychomycosis. He indicated that at the time of his presentation, he had discontinued terbinafine use about two weeks prior. At the time of admission, the patient’s blood pressure was 131/76 mmHg, heart rate was 96 beats per minute, respiratory rate was 16 breaths per minute, oxygen saturation was 96% on room air, and body temperature was 98.2 degrees Fahrenheit. Physical examination showed evidence of jaundice, scleral icterus, and a soft non-distended abdomen. Initial laboratory results at admission showed elevated total bilirubin, ALP, AST, and ALT, indicating acute hepatocyte injury leading to cholestasis (Table 1).

**Table 1:** Significant laboratory results at initial presentation. ALT, alanine aminotransferase; AST, aspartate aminotransferase; ALP, alkaline phosphatase; WBC, white blood cells; RBC, red blood cells; HGB, hemoglobin; HCT, hematocrit; MCV, mean corpuscular volume; PLT, platelets

Test	Value	Reference range
ALT	832	10-49 U/L
AST	645	0-34 U/L
Total bilirubin	32	0.3-1.2 mg/dL
ALP	1843	100-400 U/L
WBC	5.0	4.0-10.5 10^3^/uL
RBC	5.05	4.63-6.08 10^6^/uL
HGB	13.6	13.7-17.5 g/dL
HCT	43.4	40.1-51.0%
MCV	85.9	79.0-92.2 fL
PLT	291	150-400 10^3^/uL

A hepatitis panel showed no evidence of an acute viral infection. Imaging was pursued next with a chest X-ray, which exhibited no acute cardiopulmonary process. A right upper quadrant ultrasound was ordered, which showed no evidence of acute cholecystitis. The ultrasound further demonstrated a non-obstructed common bile duct and a normal echogenic liver.

**Figure 1:**
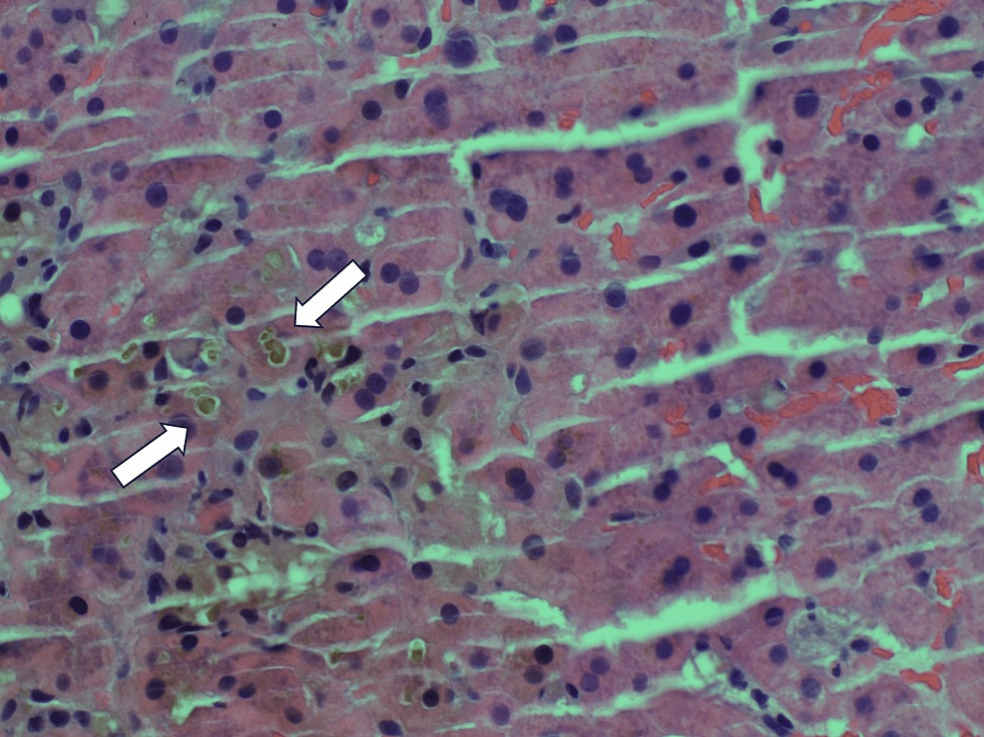
Liver biopsy showing intracellular cholestasis and bile plugs.

**Figure 2:**
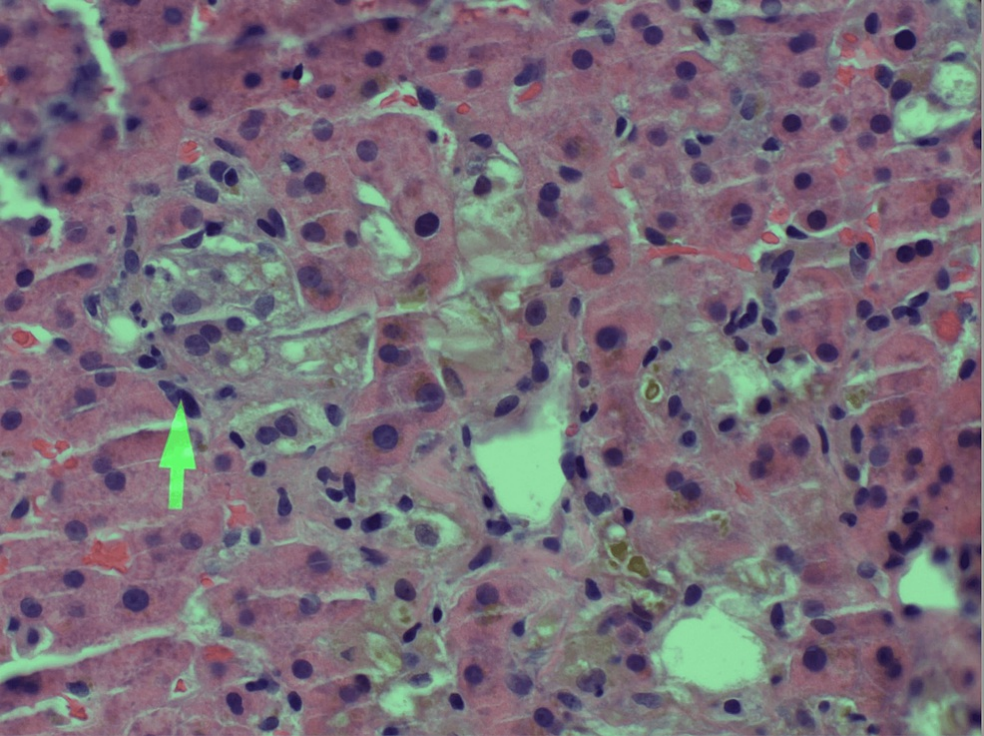
Liver biopsy showing bile granuloma.

At this point, the underlying cause of this patient's presentation was uncertain. A liver biopsy was ordered to pursue further workup and to investigate potential hepatotoxicity secondary to terbinafine use, a reported hepatotoxic agent. Three core needle biopsies of the liver were performed, which, on microscopic evaluation, showed benign hepatic parenchyma with marked intracellular cholestasis including bile plugs (Figure 1) and bile granulomas (Figure 2). Bile plugs are representative of an acute episode of cholestasis, whereas bile granulomas are representative of an acute inflammatory response. A trichrome stain showed portal tracts normal in size and shape. No evidence of periportal fibrosis or cirrhosis was found on examination. Periodic acid-Schiff (PAS) stain showed strong positivity in the hepatocytes without evidence of PAS-positive cytoplasmic globules. A special stain for iron was negative within the hepatocytes.

This patient was admitted to the hospital for 11 days, and throughout his hospital course, serial measurements of his ALT, AST, total bilirubin, direct bilirubin, and ALP levels were noted. The patient was instructed to discontinue the use of terbinafine and was treated for severe acute hepatic inflammatory response, secondary to terbinafine toxicity. His treatment regimen included prednisone 40 mg once a day for seven days, followed by a taper to 20 mg until discharge. On day 11 of his hospital course, the patient's AST and ALT levels showed moderate improvement, and he was cleared for discharge. The patient was instructed to follow up with the prison physician for further monitoring of his liver enzymes and management of acute liver injury.

## Discussion

The annual worldwide incidence of DILI is 14-19 cases per 100,000 individuals. Comparatively, liver injury secondary to the antifungal medication terbinafine has an annual global incidence of 2.5 cases per 100,000 [2,5]. Hepatotoxicity secondary to terbinafine use is a well-known reported side effect; however, it is not commonly seen in clinical practice, and, in fact, less than 1% of patients treated with terbinafine will show evidence of liver biochemistry abnormalities [6].

Confirmation of terbinafine-induced liver injury requires careful evaluation of the patient’s medication list as well as extensive workup to rule out other hepatobiliary pathologies. Terbinafine-induced hepatotoxicity presents with non-specific mixed hepatotoxic and cholestatic findings of physical examination and laboratory evaluation. With long-standing injury, patients will frequently develop a predominant cholestatic pattern [3]. Most patients will present with symptoms such as abdominal pain, malaise, scleral icterus, and severe jaundice approximately four to six weeks after initiation of terbinafine [1,2,7]. On laboratory evaluation, patients commonly present with elevations in AST, ALT, ALP, gamma-glutamyl transferase (GGT), and bilirubin levels, painting a picture of a mixed hepatic and cholestatic injury [2]. Although no specific individual finding is specific to drug-induced hepatotoxicity, liver biopsy, coupled with detailed history and laboratory findings, can help paint a clearer picture. Often, other causes of hepatobiliary pathology must be ruled out before ruling in drug-induced hepatotoxicity as it can mimic other components of a differential diagnosis including, but not limited to, viral hepatitis, autoimmune hepatitis, cholecystitis, choledocholithiasis, ascending cholangitis, primary biliary cholangitis, primary sclerosis cholangitis, bile duct stricture, pancreatic cancer, and cholangiocarcinoma [8]. Evidence highly suggestive of drug-induced liver toxicity can be seen on liver biopsy, which typically involves prominent eosinophils in inflammatory infiltrate, granulomatous hepatitis, cholestatic hepatitis, and severe acute injury with zonal sub-massive or massive hepatic necrosis. Any unusual combination of these histologic findings should warrant further evaluation for DILI [9].

Terbinafine-induced hepatotoxicity generally resolves in most cases following cessation of use. Symptoms may linger anywhere from three to six months following the discontinuation of terbinafine. Persistence of symptoms for longer than expected after discontinuation of terbinafine should prompt clinicians to evaluate for vanishing bile duct syndrome (VBDS). VBDS is a rare sequel occurring months after an episode of severe cholestatic hepatitis secondary to DILI. VBDS is marked clinically by chronic cholestasis and histologically by loss of intrahepatic bile ducts [10]. It usually occurs one to six months following the onset of hepatic injury, presenting with intense, persistent, pruritus, jaundice, fatigue, hypercholesterolemia, dyslipidemia, and severe skin xanthomata [10]. Acute liver failure secondary to terbinafine use is a rare phenomenon [1,11]. It is found that routine hepatic monitoring for patients being treated with terbinafine does not provide substantial benefit in prevention of hepatotoxicity unless the patient shows signs of clinically symptomatic liver injury [2].

The pathophysiology of terbinafine-induced hepatotoxicity is not well understood. Obstruction of bile flow by canalicular injury or other lesions of bile secretion seems to be the predominant factor in terbinafine-induced liver injury. There are two proposed mechanisms for the hepatotoxic nature of terbinafine. The first is an immune-mediated hypersensitivity reaction, and the second is a metabolic effect mediated by the formation of mono-GSH (glutathione) conjugates, which can bind to and disrupt the function of hepatobiliary proteins. The metabolic effect is supported by the lack of characteristic hypersensitivity features and the prolonged exposure to terbinafine prior to symptom onset [1,11]. One factor supporting immune-mediated injury is the presence of polymorphisms in the human leukocyte antigen (HLA) region of chromosome 6 found in genome-wide association studies of patients with terbinafine-induced liver injury [1,3]. HLA-A* 33:01 is associated with terbinafine-induced hepatotoxicity in those of European descent, whereas HLA-A* 33:03 has shown association with terbinafine-induced hepatotoxicity in those of Asian descent [3].

## Conclusions

DILI is a rare diagnosis having a low annual incidence; however, it has a symptomatic profile mimicking more common causes of hepatic injury. Therefore, clinicians must take a detailed history when examining patients with signs of hepatotoxicity so that they may be able to make a broad differential diagnosis. Clinicians should investigate recent drug changes in a patient's medical profile as well as recent acute medical treatment regimes. Only after more common causes of acute liver injury have been effectively ruled out should DILI be considered. Terbinafine is a known hepatotoxic agent used in the treatment of onychomycosis. If terbinafine is the suspected agent of hepatic injury, prompt discontinuation of its use should be considered. This case highlights the symptoms, diagnostic measures, and suspected pathophysiology of terbinafine-induced hepatotoxicity.
